# Ventricular Function in Congenital Heart Defects

**DOI:** 10.3389/fped.2016.00071

**Published:** 2016-07-14

**Authors:** Antonio F. Corno

**Affiliations:** ^1^East Midlands Congenital Heart Centre, Glenfiled Hospital, University Hospital Leicester, Leicester, UK

**Keywords:** congenital heart disease, ventricular mechanics, ventriculo-arterial coupling, modeling, HFPEF

In order to understand the physiology of the neonatal heart, one must have an understanding of both the fetal circulation and the cardiac function of the adult heart. Transitional changes occur in the neonatal period, where the function of one ventricle has important effects on the function of the contralateral ventricle (1). In the presence of congenital heart defects, the myocardium is exposed to pressure and/or volume overload with the subsequent development of hypertrophy and/or dilatation. This is further complicated by the myocardial exposure to chronic hypoxia (2).

Giovanni Biglino and Adelaide De Vecchi have organized a research topic entitled “Ventricular mechanics in congenital heart disease” in order to increase the current knowledge on the physiology of the neonatal and infant heart, particularly in relationship to the coupling of the ventricular function with the systemic and pulmonary resistance. This research topic will concentrate on myocardial function in complex congenital heart defects, including conditions with a morphologic right ventricle sustaining the systemic circulation and hearts with functionally a single ventricle.

The ventricular interactions in the presence of congenital heart defects have been primarily investigated for the past few decades using ultrasound and radioisotopes (3). This research topic will attract the contribution of researchers using advanced diagnostic techniques to investigate the myocardial function of neonates with normal hearts and with complex congenital heart defects, such as biomedical engineering, non-invasive and invasive diagnostic modalities, cardiovascular magnetic resonance imaging, finite element and statistical shape modeling, and computational fluid dynamics (Biglino et al.). This research topic will be of particular interest to those who involved in the treatment of complex congenital heart defects.

Research articles stimulated by this research topic will improve the understanding of the ventricular function in congenital heart defects and will facilitate the decision-making process related to the timing and type of intervention.

An improved knowledge of the degree of myocardial dysfunction as a result of ventricular pressure and/or volume overload due to the presence of cardiac malformations should result in improved comprehensive management strategies for each type of congenital heart defect.

## Author Contributions

The author confirms being the sole contributor of this work and approved it for publication.

## Conflict of Interest Statement

The author declares that the research was conducted in the absence of any commercial or financial relationships that could be construed as a potential conflict of interest.
